# Genetic markers associated with bone composition in Rhode Island Red laying hens

**DOI:** 10.1186/s12711-023-00818-x

**Published:** 2023-06-29

**Authors:** Moh Sallam, Peter W. Wilson, Björn Andersson, Matthias Schmutz, Cristina Benavides, Nazaret Dominguez‑Gasca, Estefania Sanchez‑Rodriguez, Alejandro B. Rodriguez‑Navarro, Ian C. Dunn, Dirk‑Jan De Koning, Martin Johnsson

**Affiliations:** 1 Swedish University of Agricultural Sciences, 75651 Uppsala, Sweden; 2 Roslin Institute, University of Edinburgh, Edinburgh, EH25 9RG Scotland UK; 3 Lohmann Breeders, 27472 Cuxhaven, Germany; 4 Departamento de Mineralogia y Petrologia, Universidad de Granada, 18002 Granada, Spain

## Abstract

**Background:**

Bone damage has welfare and economic impacts on modern commercial poultry and is known as one of the major challenges in the poultry industry. Bone damage is particularly common in laying hens and is probably due to the physiological link between bone and the egg laying process. Previous studies identified and validated quantitative trait loci (QTL) for bone strength in White Leghorn laying hens based on several measurements, including bone composition measurements on the cortex and medulla of the tibia bone. In a previous pedigree-based analysis, bone composition measurements showed heritabilities ranging from 0.18 to 0.41 and moderate to strong genetic correlations with tibia strength and density. Bone composition was measured using infrared spectroscopy and thermogravimetry. The aim of this study was to combine these bone composition measurements with genotyping data via a genome-wide association study (GWAS) to investigate genetic markers that contribute to genetic variance in bone composition in Rhode Island Red laying hens. In addition, we investigated the genetic correlations between bone composition and bone strength.

**Results:**

We found novel genetic markers that are significantly associated with cortical lipid, cortical mineral scattering, medullary organic matter, and medullary mineralization. Composition of the bone organic matter showed more significant associations than bone mineral composition. We also found interesting overlaps between the GWAS results for tibia composition traits, particularly for cortical lipid and tibia strength. Bone composition measurements by infrared spectroscopy showed more significant associations than thermogravimetry measurements. Based on the results of infrared spectroscopy, cortical lipid showed the highest genetic correlations with tibia density, which was negative (− 0.20 ± 0.04), followed by cortical CO3/PO4 (0.18 ± 0.04). Based on the results of thermogravimetry, medullary organic matter% and mineral% showed the highest genetic correlations with tibia density (− 0.25 ± 0.04 and 0.25 ± 0.04, respectively).

**Conclusions:**

This study detected novel genetic associations for bone composition traits, particularly those involving organic matter, that could be used as a basis for further molecular genetic investigations. Tibia cortical lipids displayed the strongest genetic associations of all the composition measurements, including a significantly high genetic correlation with tibia density and strength. Our results also highlighted that cortical lipid may be a key measurement for further avian bone studies.

**Supplementary Information:**

The online version contains supplementary material available at 10.1186/s12711-023-00818-x.

## Background

Laying hens have a strong tendency to suffer from bone damage (deviations or fractures), which is a major welfare challenge in the egg industry. In 1989, Gregory and Wilkins reported that ~ 30% of commercial caged layers had at least one bone fracture [1]. More recent studies from different countries showed a high incidence of bone damage, particularly in the keel bone or sternum, for chicken raised under all types of housing systems and both for brown and white laying hens: 95% in a British study [2], over 85% in a Belgian study [3], about 83% in a Swiss study [4], 25 to 70% in Danish studies [5, 6], and 27% in an Australian study [7]. All these findings indicate the high prevalence of bone damage problems, in spite of the long recognized possibility of improving bone quality via genetic approaches [8]. Birds with fractured bones tend to lay fewer eggs, eat more, and likely have a higher mortality rate [9–11]. Thus, bone damage is not only a major welfare issue but also has a clear negative economic impact.

Given that both bone and eggshell formation are processes that require large amounts of calcium, a relationship between egg laying and bone damage might be expected [12]. However, the egg laying process has several characteristics that may not genetically correlate with bone strength, and this may also vary across breeds. For example, on the one hand, pre-peak egg laying, which is negatively correlated with the onset of egg laying (in White Leghorn) and egg mass (in Rhode Island Red), showed significant negative genetic correlations with tibia strength [13]. On the other hand, post-peak egg laying showed a low and non-significant correlation with tibia strength in both breeds in the same study. Similar findings suggested a weak and non-significant relationship between egg and bone quality at 105 weeks of age in H&N Brown Nick layers [14]. Fleming et al. [15] compared bones and eggs of Lohmann Selected Leghorn (LSL) hens that had been divergently selected for high and low bone strength. Hens with a high bone strength laid more but smaller eggs than hens with a low bone strength, while eggshell strength and thickness did not differ between these two lines. These findings of Fleming et al. [15] suggest that: (1) hens could be selected for stronger bones without a negative effect on eggshell strength, and (2) it is possible to select for hens that both have stronger bones and lay more eggs (laying persistency), but with possible reductions in egg size.

Bone consists of complex composite material, which is constituted by carbonated apatite nanocrystals that mineralize an organic matrix of cross-linked collagen fibres [16]. In spite of its apparently static appearance, bone is a living dynamic tissue that is constantly accreted and remodelled by bone cells. During remodelling, old bone tissue and minerals are resorbed and new bone tissue is deposited and mineralized [17]. In the human literature, it has been reported that bone remodelling can modify bone architecture (size, shape, content, and bone cell distribution), as a response to mechanical usage, diseases, or aging [18]. In laying hens, medullary bone is resorbed during eggshell formation and deposited again during the daily egg cycle. Medullary bone is specialised bone that is deposited under the influence of estrogen to store calcium for egg shell formation [19]. However, cortical bone, which provides the most strength, can be resorbed during egg laying, resulting in progressive loss of structural bone (i.e., osteoporosis) [20]. Consequently, the mechanical properties of bone (breaking strength) in laying hens are not constant and change due to multiple factors (egg laying, physical exercise, diet, and aging) that affect bone mineralization and structure [13, 21–23].

In addition to measurements of bone strength and density, its chemical composition has been measured in laying hens to provide a more detailed picture of the biology of bones and eggs [12, 13, 24]. Li et al. [24] showed that the density of the bones of laying hens increases until the onset of egg laying, which coincides with a rise in bone carbonate, and then remains stable. In addition, measurements of the chemical composition of bone can provide an estimate of bone remodelling based on the ratio of minerals to organic matter, which gives an indication of the ongoing mineralization process and based on the ratio of carbonate to phosphates, which gives an indication of ongoing carbonate substitution.

Many avian appendicular bones are made up of an outer denser cortical component and, when the chickens are reproductively active, an inner less dense medullary component. The chemical composition (mineralization and carbonate substitution) of cortical and medullary bone varies phenotypically and genetically between hens. Rodriguez-Navarro et al. [22] reported that, in a White Leghorn breed, cortical and medullary mineralization varied within and across housing systems, due to differences in the physical activity of birds in different types of housing. Dunn et al. [13] performed a pedigree-based genetic study for tibia bone composition in White Leghorn and Rhode Island Red hens (each representing one of the common grandparents of commercial layers). Genetically, both cortical and medullary mineralization varied within and across these breeds. In addition, the heritability estimates for medullary composition measurements ranged from 0.18 to 0.41 and these measurements were genetically correlated (0.6–0.9) with tibia breaking strength. These moderate to strong heritabilities and strong genetic correlations, along with the identification [25] and subsequent validation [26] of a large quantitative trait locus (QTL) for tibia strength, suggest that adding genotyping data and running genome-wide association studies (GWAS) on bone composition traits could reveal genomic regions that contribute to multiple aspects of bone health in laying hens. The current study is the genomic follow-up of the pedigree-based study of Dunn et al. [13], with a focus on bone composition traits that have not been previously addressed. The objectives of the study were to: (1) perform GWAS to detect genetic marker associations with ~ 29 bone composition measurements in a cohort of 924 Rhode Island Red laying hens, and (2) estimate genetic correlations between tibia bone composition traits and overall tibia density and strength.

## Methods

### Animals and phenotyping

We studied a cohort of 924 Rhode Island Red hens from a pure grandparent line of Lohmann Brown commercial layers (Lohmann Breeders GmbH, Germany). The hens from four hatches were assigned to two houses (at Roslin Institute facility, Edinburgh, United Kingdom) equipped with furnished cages that each included a perch and a white egg-laying companion to enable individual recording. Birds of early hatches were assigned to one house and later hatches to the other, and within each house, birds were assigned randomly to the cages. Hens were fed ad libitum with a standard layer diet. Hens were euthanized at 68 weeks of age, weighed, and tibia bone samples were collected for further detailed bone measurements, as described in [13]. In the current study, we analysed tibia chemical composition, mineral crystallinity, and mechanical properties.

#### Tibial bone chemical composition

The chemical composition of tibia-mid-shaft cortex and medullary bone was measured by Fourier transform infrared spectroscopy (FTIR) and thermogravimetry (TGA), as described in more detail in [22]. A 1-cm section of bone that was cut from the tibia mid-shaft was selected. Then, cortical and medullary bone tissues were separated manually and homogenized by grinding. Cortical or medullary bone in powder form were analysed in reflection mode using the FTIR spectrometer (mod 6200, JASCO) equipped with an ATR unit (MIRacle Single Reflection ATR, PIKE Technologies). The infrared spectra were recorded at a 2-cm^−1^ resolution for 100 scans. The compositional parameters were determined from the peak area of the absorption bands associated with the chemical composition of bone, as shown in Table 1.

**Table 1:** Trait definition and estimates of variation coefficients, heritability, and genetic correlations with tibia density and strength

Method	Tibia			Phenotypic		Genetic correlation with	
	Bone	Trait name	Definition	Variation coefficient	h^2^ ± SE	Tibia density	Tibia breaking strength
FTIR	Cortex	Cortical PO4/Amide I	Calcium-phosphate (PO4) relative to organic matter (Amide I); calcium-phosphate and organic matter measured as FTIR area at main peak 900–1200 cm^−1^ and 1640 cm^−1^, respectively; this measurement indicates the degree of mineralization	13.43	0.08 ± 0.04	− 0.14 ± 0.04	− 0.11 ± 0.04
		Cortical CO3/PO4	Carbonate relative to calcium-phosphate(PO4); carbonate and calcium-phosphate measured as FTIR area at main peak 1415 cm^−1^ and 900–1200 cm^−1^, respectively. Carbonate peak represents carbone contribution from crystalized and non-crystalized minerals excluding carbone contribution from organic matter phase; this measurement refers to carbonate substitution and is an indicator of carbonate weight % [19]	9.13	0.07 ± 0.04	0.18 ± 0.04	0.14 ± 0.04
		Cortical CO3/Amide I	Carbonate relative to organic matter (Amide I); carbonate and organic matter measured as FTIR area at main peak 1415 cm^−1^ and 1640 cm^−1^, respectively; CO3/Amide I and PO4/Amide I together refer to bone mineralization process	6.85	0.09 ± 0.04	− 0.05 ± 0.04	− 0.04 ± 0.04
		Cortical CO3 1450/1415	Ratio of secondary carbonate and organic matter peak (1450 cm^−1^) to the main carbonate peak (1415 cm^−1^)	3.43	0.06 ± 0.04	− 0.07 ± 0.04	− 0.07 ± 0.04
		Cortical collagen maturity	Mature relative to immature collagen cross-links; mature and immature collagen measured as the FTIR area at main peak 1660 cm^−1^ and 1690 cm^−1^, respectively; this measurement is used as an indicator of the collagen maturity	60.04	0.09 ± 0.04	− 0.08 ± 0.04	− 0.10 ± 0.04
		Cortical lipid	Carbonyl group from the lipid; measured as the FTIR area at main peak 1710 cm^−1^	69.26	0.19 ± 0.05	− 0.20 ± 0.04	− 0.19 ± 0.04
	Medulla	Medullary PO4/Amide I	As in cortex. Note Amide I in medulla come from medulla bone organic matter and bone marrow as well	40.14	0.05 ± 0.03	0.08 ± 0.04	0.10 ± 0.03
		Medullary CO3/PO4	As in cortex	31.07	0.06 ± 0.04	− 0.04 ± 0.04	− 0.05 ± 0.03
		Medullary CO3/Amide I	As in cortex	29.67	0.07 ± 0.03	0.13 ± 0.04	0.15 ± 0.03
		Medulla CO3 1450/1415	As in cortex	23.41	0.02 ± 0.03	− 0.05 ± 0.04	− 0.07 ± 0.03
		Medullary collagen maturity	As in cortex	57.44	0.05 ± 0.03	− 0.02 ± 0.04	− 0.02 ± 0.03
		Medullary lipid	As in cortex	91.9	0.01 ± 0.03	− 0.02 ± 0.03	− 0.05 ± 0.03
TGA	Cortex	Cortical water %	Water weight% measured by TGA	8.51	0.00 ± 0.08	0.00 ± 0.03	0.00 ± 0.04
		Cortical OM %	Organic matter weight% measured by TGA	6.43	0.10 ± 0.04	− 0.15 ± 0.06	− 0.16 ± 0.04
		Cortical CO3%	Carbonate weight% measured by TGA	16.3	0.00 ± 0.03	0.01 ± 0.03	0.00 ± 0.04
		Cortical phosphates %	Phosphate weight% measured by TGA	2.35	0.09 ± 0.04	0.10 ± 0.04	0.11 ± 0.03
		Cortical mineral %	Minerals weight%; calculated as the sum of carbonate % and phosphate % measured by TGA	2.47	0.07 ± 0.04	0.14 ± 0.05	0.15 ± 0.04
		Cortical phosphates/OM	Phosphate weight % relative to organic matter weight % measured by TGA	7.91	0.13 ± 0.04	0.15 ± 0.04	0.15 ± 0.04
		Cortical CO3/phosphates	Carbonate weight % relative to organic matter weight % measured by TGA	16.57	0.00 ± 0.03	0.00 ± 0.03	0.00 ± 0.04
	Medulla	Medullary water %	As in cortex	16.49	0.03 ± 0.04	0.10 ± 0.04	0.08 ± 0.04
		Medullary OM %	As in cortex	19.12	0.23 ± 0.04	− 0.25 ± 0.04	− 0.20 ± 0.04
		Medullary CO3 %	As in cortex	35.72	0.04 ± 0.03	0.13 ± 0.03	0.14 ± 0.05
		Medullary phosphates %	As in cortex	31.37	0.22 ± 0.04	0.24 ± 0.04	0.20 ± 0.04
		Medullary mineral %	As in cortex	32.39	0.22 ± 0.04	0.25 ± 0.04	0.20 ± 0.04
		Medullary phosphates/OM	As in cortex	52.79	0.24 ± 0.05	0.26 ± 0.04	0.21 ± 0.04
		Medullary CO3/phosphates	As in cortex	33.41	0.04 ± 0.03	− 0.11 ± 0.03	− 0.06 ± 0.04
XRD	Cortex	Crystal scattering	Scattering degree of mineral crystals orientations within bone mineral; measured as the angular breadth of bands displayed in the intensity profile along the Debye–Scherrer ring associated with the 002 reflection of apatite mineral (Gamma scan [53]); the wider the band, the greater the scattering (less organization) in the orientation of the c-axis of apatite crystals; this is the most accurate XRD measurements	11.58	0.02 ± 0.03	0.03 ± 0.03	− 0.02 ± 0.03
		Crystal orientations	Degree of crystal orientation; it ranges from 0 (random) to 1 (completely oriented)	15.67	0.01 ± 0.03	− 0.06 ± 0.04	− 0.04 ± 0.03
		Crystal oriented fraction	Ratio of orientated to non-orientated mineral crystals; greater value means well organized crystals and smaller values means less organized crystals	25.98	0.00 ± 0.02	0.02 ± 0.03	0.02 ± 0.03

FTIR: Fourier transform infrared spectroscopy; TGA: thermogravimetry; XRD: X-ray diffraction; OM: organic matter; heritabilities (h^2^) and genetic correlations are estimated by multi-trait genomic restricted maximum likelihood; SE: standard error of the estimates; ${h}^{2}$ for tibia density: 0.50 ± 0.05; ${h}^{2}$ for tibia breaking strength: 0.46 ± 0.05

For TGA, about 25 mg of the powdered bone (cortical or medullary) were used to obtain the thermogravimetry scans (TGA). From the observed weight loss, the percentage weight of the main chemical composition of bone (water, organic, mineral, carbonate) was determined, as shown in Table 1.

Both FTIR and TGA are used to measure bone composition. For example, TGA Mineral% represents the mineral content of bone tissue; TGA OM% represents the organic matter content of bone tissue, FTIR PO4/Amide I ratio represents phosphate content (main mineral component) relative to organic matter, and TGA CO3% represents carbonate content (in the mineral part of a bone). Some differences between the FTIR and TGA methods should be noted. On the one hand, TGA measures the loss in bone sample weight at specific temperature ranges corresponding to the loss of specific components of bone during heating (water evaporation, combustion of organic matter, thermal decomposition of carbonate). On the other hand, FTIR measures the peak area of the absorption bands in the mid infrared region of different molecular components of bone (e.g. carbonate and phosphate from the minerals and amide groups from proteins). Both techniques give information on the degree of mineralization and complementary information from FTIR on collagen cross-linking and lipid content data. Although both methods can provide quantitative compositional data for bone, TGA measurements are more precise and have less variability than FTIR measurements.

#### Tibial bone mineral properties

Tibia cortical mineral crystallinity and crystal orientation were measured by X-ray diffraction (XRD), as described in more detail in [22]. A 1-cm^2^ portion of cortical bone that was cut from the tibia mid-shaft was analysed in transmission mode with a X-ray single crystal diffractometer (D8 VENTURE, Bruker) equipped with an area detector (PHOTON II) and a Mo radiation (0.2 mm collimator). Measurements related to bone mineral crystallinity (maturity) and mineral organization (apatite crystal orientation) were determined from XRD data, as described in Table 1.

#### Tibial bone mechanical properties

The mechanical properties of bones include density and breaking strength. For tibia density, the whole tibia was radiographed by X-ray, with an exposure voltage adjusted for the hen’s age. The generated X-ray images were scanned, then the tibia was delineated from the background and the mean radiographic density (pre-calibrated in mm of aluminium equivalent) of the whole bone was measured, as described in [13]. Tibia breaking strength was measured by a three-point bending test using a materials testing machine (JJ Lloyd LRX50, Sussex, UK), as described by Fleming et al. [27]. We included these mechanical traits to investigate how FTIR, TGA, and XRD measurements are genetically correlated with tibia density and strength, which are widely used to measure bone quality in poultry breeding programs. However, the genetic correlation of the tibia density and strength traits with the tibia composition traits should be interpreted with caution, since the former measures the whole tibia, while the latter measures only material from 1 cm^2^ of the tibia mid-shaft.

These different phenotyping approaches resulted in 29 traits, which are summarized with their exact definitions in Table 1.

### Genotyping

All hens were genotyped for 57,636 single nucleotide polymorphisms (SNPs) using the Illumina Infinium array. The genotyping was performed by the SNP&SEQ Technology Platform (Uppsala University, Sweden). We aligned the sequences flanking the SNPs against the GRCg6a chicken reference genome to determine the physical positions of the SNPs. One hundred and eighty-eight SNPs were removed because of their very low representation in the population and 21,230 were monomorphic in the analysed sample, leaving 36,218 SNPs for GWAS.

### Genome-wide association study and genomic heritability

For testing the association of each SNP, one-at-time, with the trait of interest, we used the following linear mixed model implemented in GEMMA version 0.98.5 [28]:

1 $$\mathbf{y}=\mathbf{X}\mathbf{b}+g \mathbf{s}\mathbf{n}\mathbf{p}+\mathbf{Z}\mathbf{h}\mathbf{e}\mathbf{n}+\mathbf{e},$$

where $\mathbf{y}$ is the standardized trait measurement; $\mathbf{X}$ is a design matrix that relates measurements $\mathbf{y}$ to the vector $\mathbf{b}$ of confounding fixed effects, including hatch, house, and the covariate of body weight; $g$ is the fixed marker effect; $\mathbf{s}\mathbf{n}\mathbf{p}$ is a vector of the SNP genotypes coded as 0, 1 and 2, respectively for common homozygous and heterozygous alleles, and rare homozygous alleles. Such coding reflects the dose of the minor allele, so, here, $g$ the marker effect is the effect of the minor allele substituting the major allele. $\mathbf{Z}$ is a design matrix that relates the measurements $\mathbf{y}$ to the vector $\mathbf{h}\mathbf{e}\mathbf{n}$ of random genetic effects. The relationship between hen effects are described by the genomic relationship matrix $\mathbf{G}$, and the variance component ratio (${\upsigma }_{\mathrm{u}}^{2}/{\upsigma }_{\mathrm{e}}^{2}$), where ${\upsigma }_{\mathrm{u}}^{2}$ is the additive genetic variance and ${\upsigma }_{\mathrm{e}}^{2}$ is the residual variance. This model can be viewed as an animal (hen) model that fits one SNP at a time as a covariate, implying that the number of animal models to be run is equal to the number of SNPs that need to be tested in the analysis, i.e. 36K SNPs in the current analysis. To facilitate such computations, GEMMA starts by setting the animal model without fitting SNPs (referred to as the null model) to estimate the variance components (${\upsigma }_{\mathrm{u}}^{2}$ and ${\upsigma }_{\mathrm{e}}^{2}$) via genomic restricted maximum likelihood (GREML), followed by adding one marker at a time to the animal model to estimate each $g$ marker effect, separately, while keeping the variance components constant.

From the variance components estimated by the GEMMA null model, the genomic-based heritability was calculated as: ${\upsigma }_{\mathrm{u}}^{2}/{\upsigma }_{\mathrm{e}}^{2}+{\upsigma }_{\mathrm{u}}^{2}$. The significance of the effects of each SNP in the GWAS model was tested using the Wald test statistic, i.e. the square of each $g$ deviated from the mean of the null hypothesis ($\upmu =0$), divided by the standard deviation $({\upsigma }_{\widehat{g}\mathrm{GWAS}})$ of the GWAS SNP effects: ${({g}_{\mathrm{GWAS}})}^{2}/({\upsigma }_{g\mathrm{GWAS}})$. Therefore, the p-values cited in the text refer to “Wald Test P-values”. We used the Bonferroni correction to define the p-value significance threshold, by dividing the 0.05 error fraction by the number of SNPs tested: 0.05/36218 = 1.38 × 10^–6^, and the p-value of 10^–5^ as a suggestive threshold. Possible inflation of p-values was inspected using quantile–quantile plots of the observed − log10(p-value) against the expected − log10(p-value).

### Genetic correlations

Genetic correlations between traits were estimated by multi-trait genomic restricted maximum likelihood, implemented in GEMMA [29]. For the multi-trait analysis, we combined all FTIR traits with tibia density and strength traits into one group, and all TGA traits with tibia density and strength traits into a second group. All traits were standardized prior to the analysis. The covariates in the multi-trait analysis were the same as in the single-trait analyses.

### Partial phenotypic correlations

All traits included in the study were standardized, then regressed on body weight. Residuals resulting from such a regression (i.e. traits adjusted for body weight) were the inputs to calculate the partial phenotypic correlation between all traits using the “stats” R package.

### Linkage disequilibrium

In order to investigate the potential correlations between the genotypes of significant SNPs (QTL), we computed the pairwise linkage disequilibrium between all genetic markers that showed an association with a p-value < 10^–4^. In addition, we calculated the local linkage disequilibrium that existed between each significant SNP (lead SNP) and the other SNPs located 3 Mbp upstream and downstream of the lead SNP. The squared correlation coefficients (${\mathrm{r}}^{2}$), as implemented in the “genetics” R package [30], were used for linkage disequilibrium statistics: ${\mathrm{r}}^{2}=({{\mathrm{P}}_{AB}-{\mathrm{P}}_{A}{\mathrm{P}}_{B})}^{2}/({\mathrm{P}}_{A}{\mathrm{P}}_{B}{\mathrm{P}}_{a}{\mathrm{P}}_{b})$, where $\mathrm{P}$ is the frequency, $A/a$ is the first/second allele at one locus and $B/b$ is the first/second allele at another locus. ${\mathrm{P}}_{AB}$ is the frequency of genotypes (haplotype) that have alleles $A$ and $B$ at two different loci.

### Overlap of genome-wide significant association results with Ensembl genes and known QTL

The significant and suggestive SNP positions for each trait were compared to the Chicken Ensembl Gene (release 106-Apr 2022) annotation. Significant and suggestive SNPs that matched with annotated genes were considered as candidate genes for the corresponding trait. In addition, we investigated the overlap between the GWAS results and 16,271 QTL from 367 publications representing 442 traits, which are curated in the Chicken Quantitative Trait Locus Database (Chicken QTLdb: animalgenome.org) using the "gallo" R package [31].

## Results

Phenotyping of bone composition using the FTIR and TGA methods reflects distinct variations in bone minerals and organic matter (for variation coefficients, see Table 1). The FTIR method provides measures of organic matter, lipid, and collagen maturity, and these measures showed more variability than measures of mineral contents. The TGA method includes only one measure of organic matter, which shows less variability than mineral content. For both the FTIR and TGA methods, the measures of mineral content were more variable in the medullary than in the cortical bones.

We found several novel genetic markers that were significantly associated with different bone properties (chemical compositional and structural parameters), as determined by the FTIR, TGA, and XRD analytical techniques, e.g. the amount of lipid in cortical bone, the orientation of apatite crystals in cortical bone, and the organic and mineral content of medullary bone. Interestingly, we also observed some overlap in the GWAS results between the tibia bone compositional traits. We report the genomic heritability of tibia (cortex and medullary) composition traits, in addition to their genetic correlations with tibia density and strength.

### Genome-wide association results

**Fig. 1:**
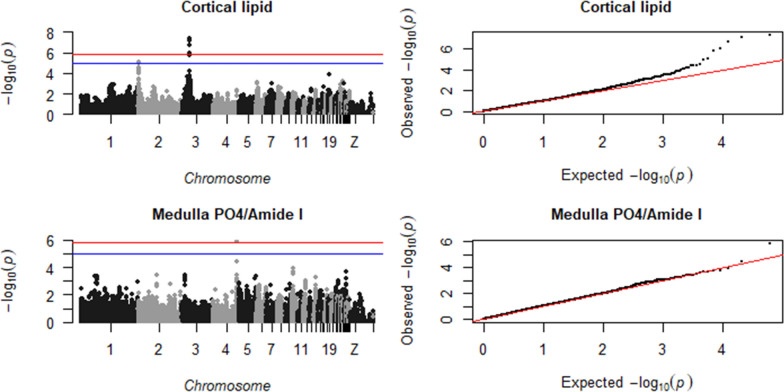
Manhattan plot (left), showing the −log10(p-value) for each SNP, and QQ plot (right), showing the observed − log10(p-value) plotted against the expected − log10(p-value), for tibial cortical lipid and medulla PO4/Amide I. The red line is the significance threshold of 1.38 × 10^–6^, and the blue is a suggestive threshold of 10^–5^

**Fig. 2:**
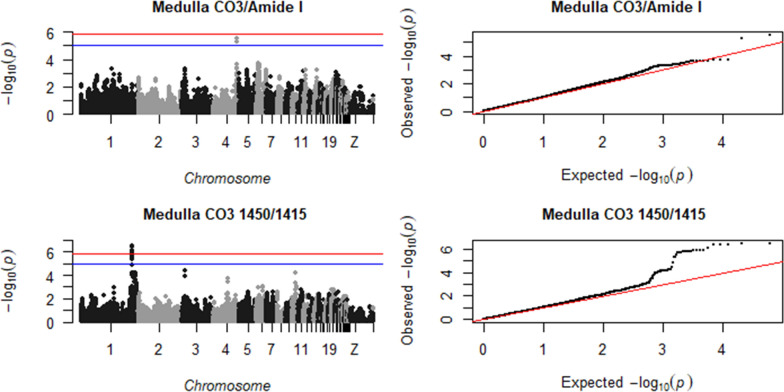
Manhattan plot (left), showing the −log10(p-value) for each SNP, and QQ plot (right), showing the observed −log10(p-value) plotted against the expected −log10(p-value), for tibial medulla CO3/Amide I and medulla CO3 1450/1415. The red line is the significance threshold of 1.38 × 10^–6^, and the blue is a suggestive threshold of 10^–5^

**Fig. 3:**
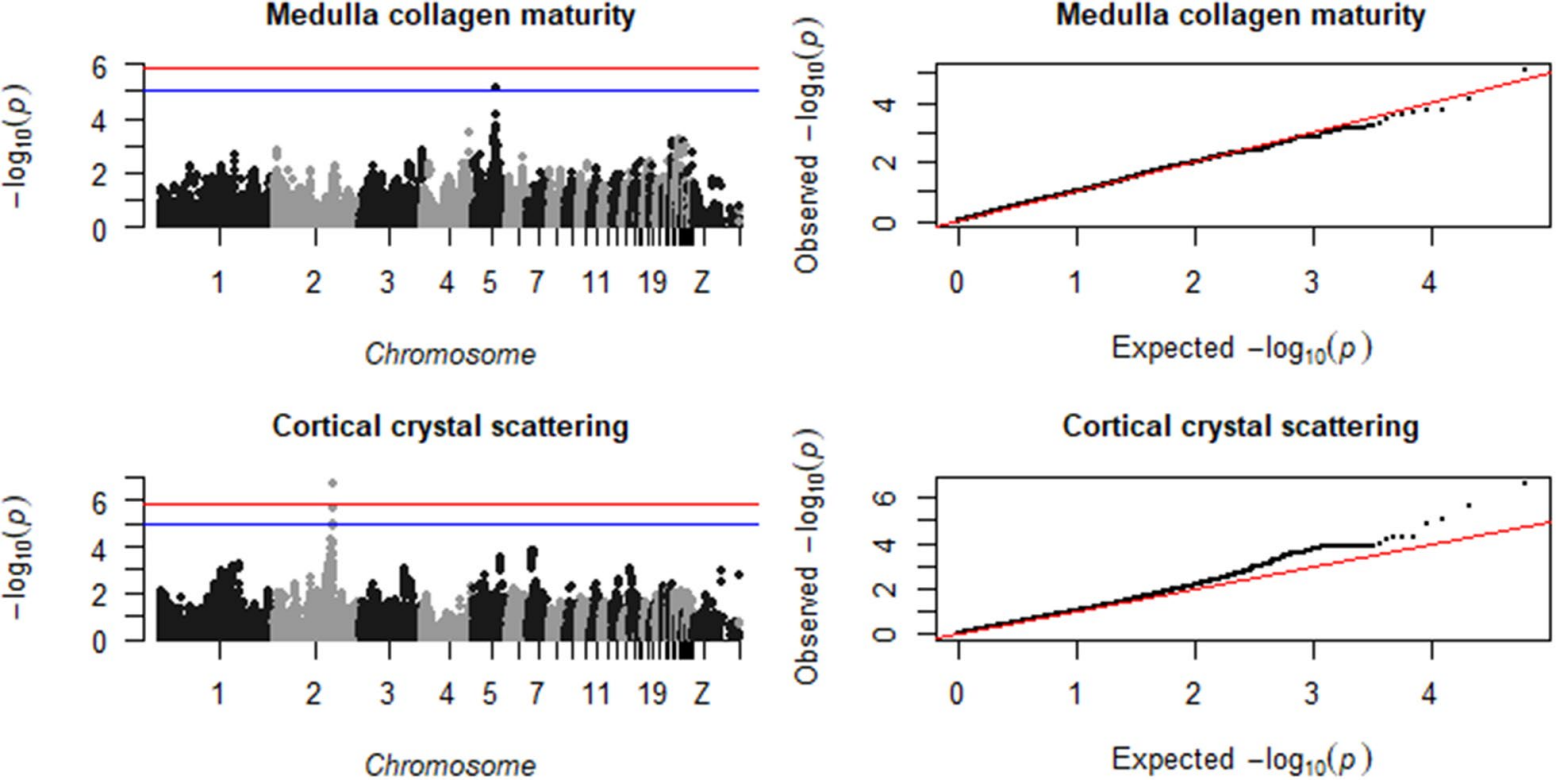
Manhattan plot (left), showing the −log10(p-value) for each SNP, and QQ plot (right), showing the observed −log10(p-value) plotted against the expected −log10(p-value), for tibial medulla collagen maturity and cortical crystal scattering. The red line is the significance threshold of 1.38 × 10^–6^, and the blue is a suggestive threshold of 10^–5^

**Fig. 4:**
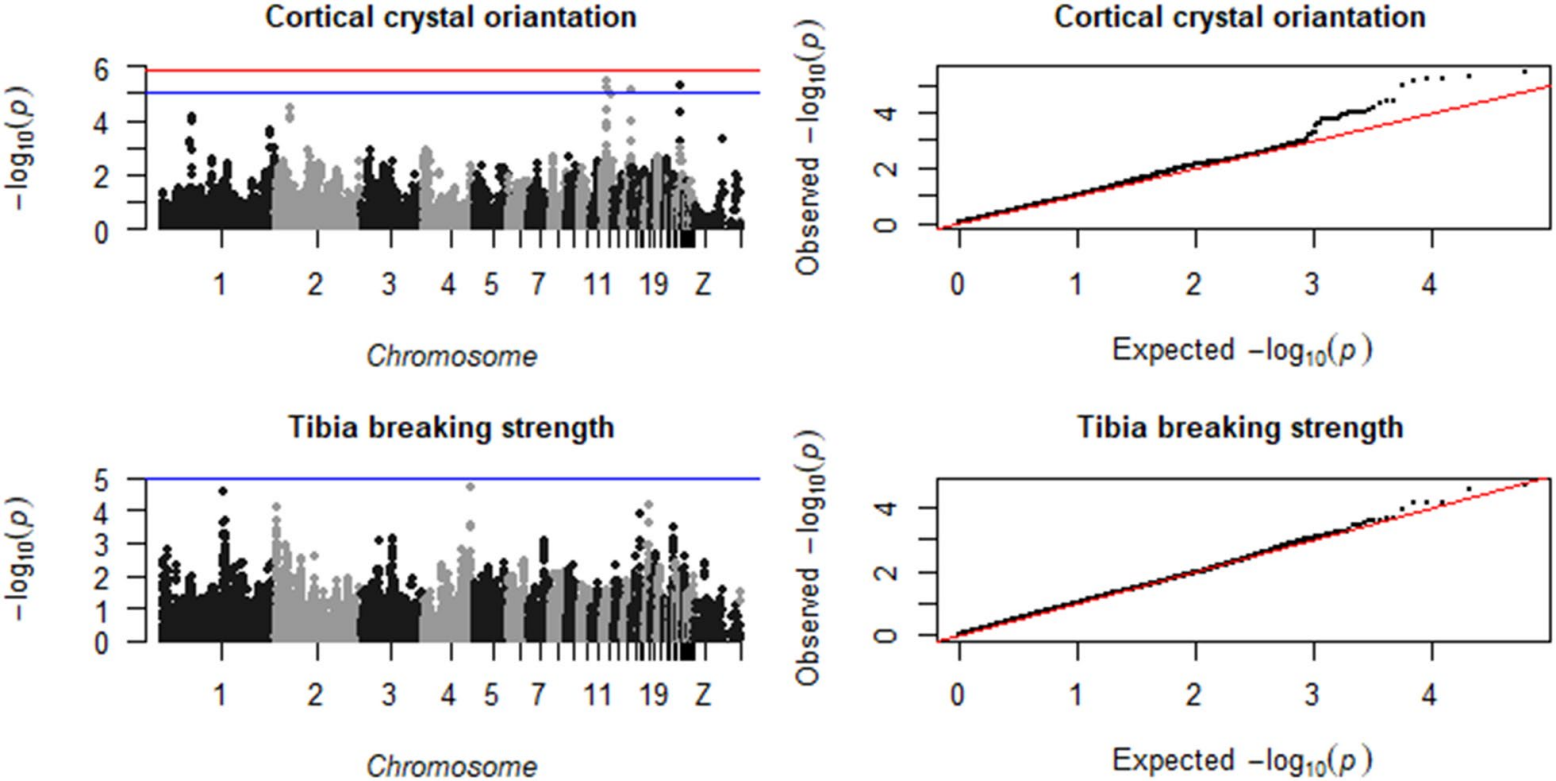
Manhattan plot (left), showing the –log10(p-value) for each SNP, and QQ plot (right), showing the observed − log10(p-value) plotted against the expected − log10(p-value), for tibial cortical crystal orientation and breaking strength. The red line is the significance threshold of 1.38 × 10^–6^, and the blue is a suggestive threshold of 10^–5^

**Fig. 5:**
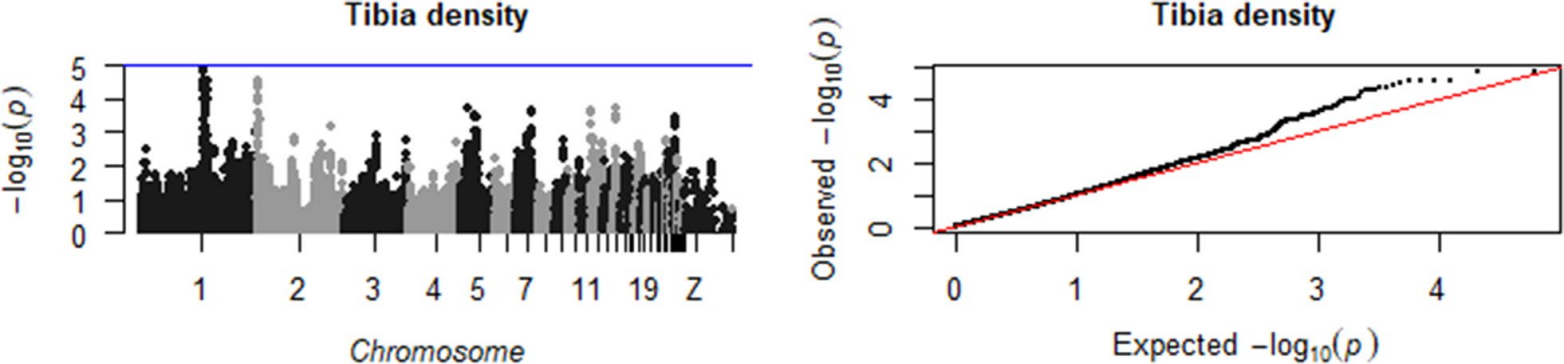
Manhattan plot (left), showing the −log10(p-value) for each SNP, and QQ plot (right), showing the observed −log10(p-value) plotted against the expected −log10(p-value), for tibia density. The red line is the significance threshold of 1.38 × 10^–6^, and the blue is a suggestive threshold of 10^–5^

Our results revealed 28 SNPs (on chromosomes 1, 2, 3, and 5) that were found to be associated with tibia organic matter composition and 11 SNPs (on chromosomes 2, 4, 12, 14, and 25) that were associated with tibia mineral composition (Table 2). Seven (out of 29) traits showed significant and suggestive associations: FTIR cortical lipid (on chromosomes 2 and 3), FTIR medullary PO4/Amide I (on chromosome 4), FTIR medullary CO3/Amide I (on chromosome 4), medullary CO3 1450/1415 (on chromosome 1), FTIR medullary collagen maturity or cross-linking (on chromosome 5), cortical crystal scattering (on chromosome 2) and cortical crystal orientation (on chromosomes 12, 14 and 25). Figures 1, 2, 3, 4, 5 show the Manhattan and QQ plots for these seven traits and for the tibia density and strength traits. Table 2 shows the position of the SNPs, their estimated effects, and the p-values of the significant and suggestive associations. Associations with a p-value < 10^–4^ and > 10^–5^ are reported in Additional file 1: Table S1.

**Table 2:** Significant and suggestive SNPs by trait, with their positions, estimated effect, p-value, and annotation in the Chicken Ensembl release 106—Apr 2022

Trait	OM/Min	SNP position		Minor allele	Effect size^a^	P-value^b^	Sig/Sug	Closest genes^c^	Gene name
		Chr	bp						
FTIR cortical lipid	OM	2	2,585,350	A	− 0.27	8.6E−06	Sug	*ENSGALG00000042657*	*WNT3A Wnt family member 3A*
	OM	3	27,204,115	G	0.30	7.6E−08	Sig	*ENSGALG00000010020*	*TTC7A tetratricopeptide repeat domain 7A, located close to CALM2 calmodulin 2*
	OM	3	27,351,346	C	0.29	1.9E−07	Sig	*ENSGALG00000010026*	*PPP1CB protein phosphatase 1 catalytic subunit beta*
	OM	3	27,434,588	G	0.31	4.5E−08	Sig	uncharacterized protein coding	
	OM	3	27,548,492	A	0.29	1.0E−06	Sig	*ENSGALG00000010039*	BRE brain and reproductive organ-expressed
	OM	3	27,648,733	G	0.29	1.7E−06	Sug	*ENSGALG00000010039*	BRE brain and reproductive organ-expressed
FTIR medullary PO4/Amide I	Min	4	83,057,186	A	0.26	1.3E−06	Sig	Non coding	
	Min	4	83,057,186	A	0.26	3.1E−06	Sug	Non coding	
	Min	4	83,154,195	G	0.23	5.1E−06	Sug	Uncharacterized protein coding	
FTIR medulla CO3 1450/1415	OM	1	175,454,102	C	0.24	1.5E−06	Sug	*ENSGALG00000042339*	Uncharacterized protein coding
	OM	1	175,547,167	G	0.23	3.2E−06	Sug	*ENSGALG00000042339*	Uncharacterized protein coding
	OM	1	175,579,717	A	0.24	1.6E−06	Sug	non coding	
	OM	1	175,604,918	A	0.24	1.6E−06	Sug	*ENSGALG00000017068*	Uncharacterized protein coding
	OM	1	175,622,214	A	0.24	1.6E−06	Sug	*ENSGALG00000017068*	Uncharacterized protein coding
	OM	1	175,631,230	G	0.24	1.8E−06	Sug	*ENSGALG00000017068*	Uncharacterized protein coding
	OM	1	175,660,267	A	0.24	1,8E−06	Sug	Non coding	
	OM	1	175,680,614	G	0.23	4.3E−06	Sug	Non coding	
	OM	1	175,696,576	A	0.24	1.8E−06	Sug	*ENSGALG00000047002*	*lncRNA*
	OM	1	175,750,307	G	0.23	5.0E−06	Sug	*ENSGALG00000017070*	*PDS5B PDS5 cohesin associated factor B*
	OM	1	175,788,839	A	0.24	1.2E−06	Sig	*ENSGALG00000017070*	*PDS5B PDS5 cohesin associated factor B*
	OM	1	175,799,752	G	0.24	1.2E−06	Sig	*ENSGALG00000017070*	*PDS5B PDS5 cohesin associated factor B*
	OM	1	175,836,437	G	0.24	1.2E−06	Sig	*ENSGALG00000053592*	*lncRNA*
	OM	1	175,934,229	A	0.24	1.5E−06	Sug	*ENSGALG00000017073*	*BRCA2 DNA repair associated*
	OM	1	176,026,352	A	0.24	1.2E–−06	Sig	*ENSGALG00000017075*	*FRY microtubule binding protein*
	OM	1	176,301,701	A	0.25	3.6E−07	Sig	*ENSGALG00000017076*	*B3GLCT beta 3-glucosyltransferase*
	OM	1	176,311,780	G	0.26	3.1E−07	Sig	*ENSGALG00000017076*	*B3GLCT beta 3-glucosyltransferase*
	OM	1	176,597,999	G	0.25	7.6E−07	Sig	*ENSGALG00000017083*	*KATNAL1 katanin catalytic subunit A1 like 1 located close to HSPH1 heat shock protein family H*
	OM	1	176,670,270	C	0.26	3.6E−07	Sig	*ENSGALG00000017084*	*UBL3 ubiquitin like 3*
	OM	1	176,699,327	G	0.26	3.8E−07	Sig	*ENSGALG00000017084*	*UBL3 ubiquitin like 3*
	OM	1	176,773,112	A	0.26	2.9E−07	Sig	non coding	
Medullary collagen maturity	OM	5	37,871,918	G	− 0.22	8.0E−06	Sug	*ENSGALG00000010192*	*FBXO33 F-box protein 33*
Cortical crystal scattering	Min	2	102,786,582	A	− 0.25	2.2E−07	Sig	Non coding	
	Min	2	102,836,922	A	− 0.22	2.1E−06	Sug	*ENSGALG00000014982*	Protein coding
	Min	2	102,886,544	A	− 0.21	1.0E−05	Sug	Non coding	
Cortical crystal orientations	Min	12	8,004,788	G	− 0.43	5.9E−06	Sug	*ENSGALG00000005400*	*CACNA2D3 calcium voltage-gated channel auxiliary subunit alpha2delta 3*
	Min	12	8,014,900	A	− 0.44	3.5E−06	Sug	*ENSGALG00000005400*	*CACNA2D3 calcium voltage-gated channel auxiliary subunit alpha2delta 3*
	Min	12	8,021,298	G	− 0.43	6.4E−06	Sug	*ENSGALG00000005400*	*CACNA2D3 calcium voltage-gated channel auxiliary subunit alpha2delta 3*
	Min	14	14,169,463	G	− 0.32	7.6E−06	Sug	*ENSGALG00000009297*	*TELO2 telomere maintenance 2*
	Min	25	2,894,535	G	− 0.52	5.0E−06	Sug	*ENSGALG00000024094*	*UBAP2L ubiquitin associated protein 2 like*

OM: Organic matter; Min: Mineral component of bone; Sig: significant with p-value < 1.38*10^−6^; Sug: suggestive with p-value < 10^−5^ and > 1.38*10^−6^

^a^All traits were standardized (with zero mean and one standard deviation) to facilitate effect size interpretation; effect here is the effect of the minor allele or the effect of the major allele with an opposite sign

^b^Wald test P-value

^c^Identified by annotating marker position to Chicken Ensembl Gene

### Overlap of genome-wide association results between tibia traits

We detected several SNPs that were associated with more than one trait (at a p-value < 10^–4^; Table 3). For example, the SNP at position 111,607,488 bp on chromosome 1 was associated with tibia density, medullary mineral%, and medullary phosphates/OM, and all the effects estimated for this SNP were positive. In another example, the SNP at position 111,721,984 bp on chromosome 1 was negatively associated with medullary OM%, but positively associated with medullary mineral%, medullary phosphates%, and medullary phosphates/OM. Similarly, on chromosome 2, the SNPs at positions 3,045,317 and 3,055,823 bp were negatively associated with cortical lipid, but positively associated with tibia density and strength, which is also consistent with the negative genetic correlation that we estimated between cortical lipid and strength using GREML. On chromosome 3, three SNPs (footnote 5, Table 3) were associated with cortical lipid (p*-*value < 10^–5^), which is one of the FTIR measurements, and also with cortical OM% (p*-*value < 10^–4^), which is one of the TGA measurements, suggesting that both measurement methods capture a similar genetic component. The other overlaps between associations (p-value < 10^–5^) detected in the current study with associations or QTL in the Chicken QTLdb (animalgenome.org) are listed in Additional file 2: Table S2.

**Table 3:** Overlap of GWAS results across tibia traits, with their positions, estimated marker effects and p-values

SNP position		Minor allele	Traits	Effect size^a^	P-value^b^
Chr	bp				
1	107,054,728	A	Tibia strength, Tibia density^c^	0.42, 0.40	2.7E−05, 3.7E−05
1	109,647,034	A	Medullary mineral %, Medullary phosphates/OM^c^	0.22, 0.24	8.0E−05, 2.3E−05
1	109,663,826	A	Medullary mineral %, Medullary phosphates/OM^c^	0.22, 0.24	8.0E−05, 2.3E−05
1	109,711,929	A	Medullary mineral %, Medullary phosphates/OM^c^	0.22, 0.24	8.0E−05, 2.3E−05
1	109,874,806	G	Medullary mineral %, Medullary phosphates/OM^c^	0.22, 0.24	9.0E−05, 2.7E−05
1	110,022,517	A	Medullary mineral %, Medullary phosphates/OM^c^	0.22, 0.24	5.6E−05, 1.5E−05
1	111,607,488	G	Tibia density, Medullary mineral %, Medullary phosphates/OM^c^	0.22, 0.22, 0.22	5.7E−05, 9.9E−05, 9.5E−05
1	111,673,836	A	Tibia density, TGA Medullary OM %^d^	0.24, − 0.23	2.7E−05, 7.7E−05
1	111,721,984	G	TGA Medullary OM %, Medullary mineral %, Medullary phosphates %, Medullary phosphates/OM^d^	− 0.23, 0.23, 0.22, 0.23	3.3E−05, 3.0E−05, 4.7E−05, 3.3E−05
1	111,798,225	A	Medullary mineral %, Medullary phosphates/OM^c^	0.25, 0.25	4.5E−05, 3.9E−05
1	111,807,107	A	TGA Medullary OM %, Medullary mineral %,Medullary phosphates %, Medullary phosphates/OM^d^	− 0.22, 0.24, 0.23, 0.25	8.9E−05, 1.9E−05, 4.5E−05, 1.9E−05
1	111,962,126	G	TGA Medullary OM %, Medullary mineral %^d^	− 0.22, 0.21	5.7E−05, 8.8E−05
1	113,308,308	A	TGA Medullary OM %, Medullary mineral %, Medullary phosphates/OM^d^	− 0.22, 0.21	5.7E−05, 8.8E−05
2	2,629,649	A	Tibia density, Cortical lipid^d^	0.24, − 0.26	8.8E-05, 3.2E−05
2	2,684,066	G	Tibia density, Cortical lipid^d^	0.24, − 0.26	8.8E−05, 3.2E−05
2	2,766,721	G	Tibia density, Cortical lipid^d^	0.25, − 0.25	5.0E−05, 3.9E−05
2	2,862,519	C	Tibia density, Cortical lipid^d^	0.25, − 0.25	5.0E−05, 3.9E−05
2	3,045,317	A	Tibia strength, Tibia density,Cortical lipid^d^	0.25, 0.25, − 0.24	7.6E−05, 2.7E−05, 7.9E−05
2	3,055,823	C	Tibia strength, Tibia density,Cortical lipid^d^	0.25, 0.25, − 0.24	7.6E−05, 2.7E−05, 7.9E−05
2	99,042,312	A	Cortical PO4/Amide I, TGA Cortical OM %^c,e^	0.30, 0.29	2.0E−05, 5.2E−05
3	27,204,115	G	Cortical lipid, TGA Cortical OM %^c,e^	0.30, 0.23	7.6E−08, 2.1E−05
3	27,351,346	C	Cortical lipid, TGA Cortical OM %^c,e^	0.29, 0.21	1.9E−07, 5.0E−05
3	27,434,588	G	Cortical lipid, TGA Cortical OM %^c,e^	0.31, 0.22	4.5E−08, 6.9E−05
4	83,057,186	A	Medullary PO4/Amide I, Medullary CO3/Amide I^c^	0.26, 0.26	1.3E−06, 3.1E−06
4	83,154,195	G	Medullary PO4/Amide I, Medullary CO3/Amide I^c^	0.21, 0.23	4.0E−05, 5.1E−06

^a^All traits were standardized (with zero mean and one standard deviation) to facilitate effect size interpretation

^b^Wald test P-value

^c^Cases of genetic marker affects different traits and the effects have the same direction

^d^Cases of genetic marker affects different traits and the effects have opposite directions

^e^Cases of overlapping between FTIR and TGA measurements

### Linkage disequilibrium

**Fig. 6:**
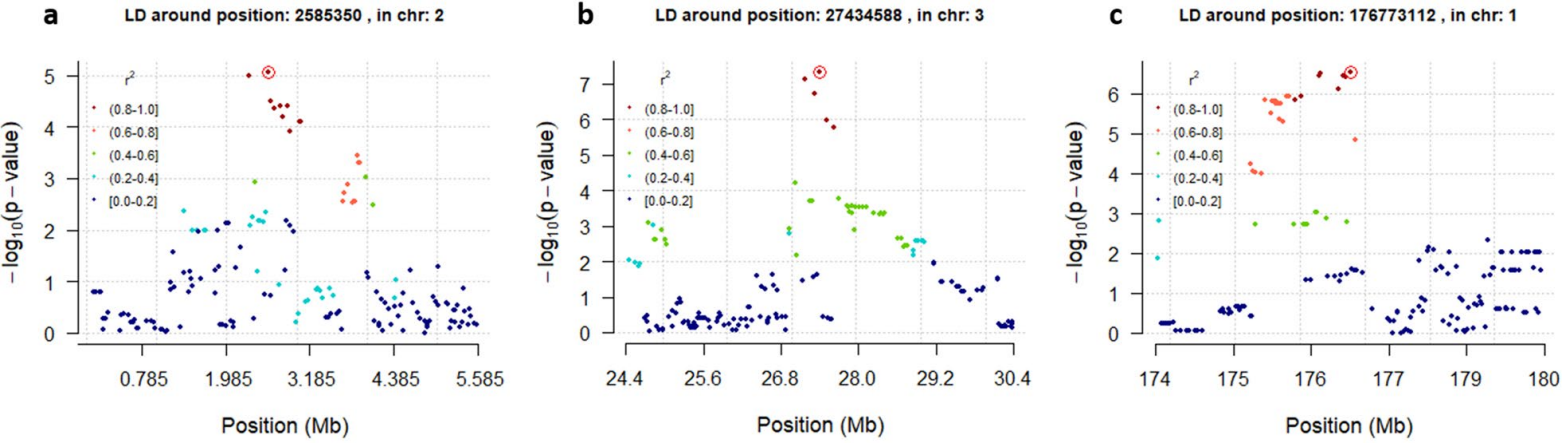
Linkage disequilibrium (LD) plots, showing the local LD structure 3 Mb upstream and downstream the most significant SNPs for cortical lipid on chromosomes 2 and 3 (a: left and b: center), and of medullary CO3 1450/1415 on chromosome 1 (c: right). Each point represents a SNP. The y-axis indicates the significance of each SNP [− 10log(p-value)], while the color coding indicates the level of LD with the most significant SNP (encircled point)

Linkage disequilibrium results showed that significant/suggestive SNPs could be correlated within chromosomes but not across chromosomes, as shown in Additional file 3: Fig. S1. The significant SNPs (lead SNPs) showed strong correlations with closely located SNPs and lower correlations with the distantly located SNPs (Local linkage disequilibrium: Fig. 6). Multiple significant SNPs that are in high linkage disequilibrium on the same chromosome likely represent effects of the same QTL or a cluster of tightly linked QTL, which is the case for the cortical lipid lead SNP and the surrounding ones on chromosomes 2 or 3 (Plot a or b: Fig. 6). This is also likely for the lead SNP associated with FTIR medullary CO3 1450/1415 and the surrounding ones on chromosome 1 (Plot c in Fig. 6).

### Genomic heritability

Estimates of genomic heritability for bone composition traits were low to moderate (Table 1). In general, the heritability estimates for FTIR bone composition traits were lower than 0.09, except for cortical lipid, which was equal to 0.2. The heritability estimates for TGA cortical bone traits ranged from 0 to 0.09, with the exception of a heritability estimate of 0.10 for cortical OM% and of 0.13 for cortical Phosphates/OM. The heritability estimates for TGA medullary bone traits were equal to 0.23 for Medullary OM%, 0.24 for Medullary Phosphates/OM, and 0.22 for Mineral% and Phosphate%, while heritability estimates were lower than 0.04 for the other traits.

### Genetic correlations of FTIR measurements

Among FTIR measurements (Table 4), estimates of genetic correlations between cortical and medullary traits range from 0.07 to − 0.09. The two medullary traits that are related to the degree of mineralization (PO4/Amide I and CO3/Amide I), showed a weak genetic correlation estimate of 0.07 ± 0.03. The two cortical mineralization traits (cortical PO4/Amide I and cortical CO3/Amide I) were genetically positively correlated, and they also showed a positive genetic correlation estimate with cortical lipid (0.14 ± 0.04 and 0.09 ± 0.04, respectively).

**Table 4:** Estimate of additive genetic variance (diagonal), genetic correlation (below diagonal) with standard errors, in addition to the partial phenotypic correlations (above diagonal), for FTIR traits

	Tibia density	Tibia breaking strain	CT PO4/Amide I	CT CO3/PO4	CT CO3/Amide I	CT CO3 1450/1415	CT collagen maturity	CT lipid	MD PO4/Amide I	MD CO3/PO4	MD CO3/Amide I	MD CO3 1450/1415	MD collagen maturity	MD lipid
Tibia density	0.44 ± 0.06	0.67	− 0.17	0.26	0.01	− 0.17	− 0.17	− 0.32	0.13	− 0.02	0.21	− 0.12	− 0.05	− 0.09
Tibia breaking strain	0.33 ± 0.05	0.42 ± 0.06	− 0.19	0.25	− 0.04	− 0.16	− 0.19	− 0.32	0.14	− 0.05	0.21	− 0.08	− 0.08	− 0.07
CT PO4/Amide I	− 0.14 ± 0.04	− 0.11 ± 0.04	0.12 ± 0.05	− 0.86	0.8	− 0.29	0.27	0.39	0	− 0.05	− 0.04	0.05	0.15	0.08
CT CO3/PO4	0.18 ± 0.04	0.13 ± 0.04	− 0.11 ± 0.05	0.11 ± 0.05	− 0.42	0.12	− 0.24	− 0.29	0.07	0.01	0.11	− 0.08	− 0.12	− 0.08
CT CO3/Amide I	− 0.05 ± 0.04	− 0.04 ± 0.04	0.10 ± 0.05	− 0.06 ± 0.04	0.11 ± 0.05	− 0.44	0.24	0.32	0.1	− 0.09	0.06	0	0.14	0.05
CT CO3 1450/1415	− 0.07 ± 0.04	− 0.06 ± 0.04	0.01 ± 0.03	− 0.04 ± 0.03	− 0.03 ± 0.04	0.08 ± 0.04	0.09	0.24	− 0.13	0.07	− 0.15	0.02	− 0.09	− 0.02
CT collagen maturity	− 0.08 ± 0.04	− 0.10 ± 0.04	0.05 ± 0.04	− 0.05 ± 0.03	0.03 ± 0.03	0.04 ± 0.04	0.10 ± 0.04	0.53	0.01	− 0.03	− 0.03	− 0.07	0.07	− 0.07
CT lipid	− 0.20 ± 0.04	− 0.19 ± 0.04	0.14 ± 0.04	− 0.13 ± 0.04	0.09 ± 0.04	0.06 ± 0.04	0.10 ± 0.04	0.20 ± 0.05	0.01	− 0.02	− 0.08	0.08	0.09	0.1
MD PO4/Amide I	0.08 ± 0.04	0.11 ± 0.04	− 0.04 ± 0.03	0.05 ± 0.03	− 0.01 ± 0.03	− 0.01 ± 0.03	− 0.01 ± 0.03	− 0.06 ± 0.04	0.06 ± 0.03	− 0.59	0.82	− 0.22	− 0.11	− 0.07
MD CO3/PO4	− 0.04 ± 0.04	− 0.05 ± 0.04	0.00 ± 0.03	− 0.01 ± 0.03	− 0.01 ± 0.04	0.00 ± 0.03	0.00 ± 0.04	0.01 ± 0.04	− 0.05 ± 0.03	0.07 ± 0.05	− 0.27	0	0	− 0.17
MD CO3/Amide I	0.13 ± 0.04	0.15 ± 0.04	− 0.06 ± 0.03	0.07 ± 0.03	− 0.02 ± 0.03	− 0.02 ± 0.03	− 0.04 ± 0.03	− 0.09 ± 0.03	0.07 ± 0.03	− 0.03 ± 0.03	0.09 ± 0.03	− 0.36	− 0.18	− 0.31
MD CO3 1450/1415	− 0.05 ± 0.04	− 0.06 ± 0.04	0.01 ± 0.04	− 0.01 ± 0.03	− 0.01 ± 0.04	0.03 ± 0.04	0.01 ± 0.04	0.04 ± 0.04	− 0.03 ± 0.04	0.03 ± 0.04	− 0.03 ± 0.03	0.05 ± 0.05	0.09	0.74
MD collagen maturity	− 0.02 ± 0.04	− 0.02 ± 0.04	0.04 ± 0.03	− 0.02 ± 0.03	0.03 ± 0.03	0.00 ± 0.03	0.05 ± 0.03	0.04 ± 0.04	0.00 ± 0.03	0.02 ± 0.03	− 0.01 ± 0.03	0.01 ± 0.03	0.08 ± 0.05	0.12
MD lipid	− 0.02 ± 0.03	− 0.04 ± 0.03	0.02 ± 0.04	− 0.02 ± 0.03	0.01 ± 0.04	0.02 ± 0.04	0.02 ± 0.03	0.04 ± 0.04	− 0.01 ± 0.04	0.00 ± 0.04	− 0.02 ± 0.02	0.02 ± 0.05	0.01 ± 0.02	0.03 ± 0.06

FTIR: Fourier transform infrared spectroscopy; CT: cortical bone; MD: medullary bone; OM: organic matter

Genetic correlations are estimated by multi-trait genomic restricted maximum likelihood; partial phenotypic correlations: phenotypes (adjusted for body weight) correlations

Both cortical and medullary bone compositional traits contribute (either positively or negatively) to tibia density and strength, but the contributions of cortical bone traits are greater than those of medullary bone traits. Cortical lipid displayed the highest genetic correlation estimate with tibia density and strength (− 0.20 ± 0.04), followed by cortical CO3/PO4 (0.18 ± 0.04). Cortical mineralization traits showed negative genetic correlation estimates with tibia density and strength traits, while the same mineralization traits in medullary bone showed positive genetic correlation estimates with tibia density and strength. Cortical CO3/PO4 (related to carbonate substitution in the mineral) showed positive genetic correlation estimates with tibia density and strength, while in medullary bone, the equivalent measurement showed negative or zero genetic correlation estimates with tibia density and strength.

### Genetic correlations of TGA measurements

Among the TGA traits (Table 5), cortical and medullary organic matter were estimated to have a positive genetic correlation, and these two traits also had inverse estimates of genetic correlations with all cortical and medullary mineral traits, except with the measure of carbonate substitution. Carbonate substitution (CO3/Phosphates) in the medullary bone is associated with cortical and medullary organic matter accumulation.

**Table 5:** Estimate of additive genetic variance (diagonal), genetic correlation (below diagonal) with standard errors, in addition to the partial phenotypic correlations (above diagonal), for TGA traits

	Tibia density	Tibia breaking strain	CT OM %	CT CO3%	CT phosphates %	CT mineral %	CT phosphates/OM	CT CO3/phosphates	MD OM %	MD CO3%	MD phosphates %	MD mineral %	MD phosphates/OM	MD CO3/phosphates
Tibia density	0.41 ± 0.06	0.67	− 0.3	0.1	0.16	0.24	0.3	0.08	− 0.51	0.31	0.51	0.51	0.51	− 0.16
Tibia breaking strain	0.32 ± 0.05	0.43 ± 0.06	− 0.31	0.05	0.16	0.24	0.32	0.02	− 0.34	0.2	0.34	0.33	0.33	− 0.09
CT OM %	− 0.15 ± 0.06	− 0.16 ± 0.04	0.11 ± 0.02	0	− 0.7	− 0.66	− 0.87	0.09	0.22	− 0.14	− 0.21	− 0.21	− 0.21	0.06
CT CO3%	0.01 ± 0.03	0.01 ± 0.03	0.00 ± 0.02	0.00 ± 0.01	− 0.2	0.15	− 0.03	0.96	− 0.01	0.12	0.01	0.01	0.03	0.1
CT phosphates %	0.10 ± 0.04	0.10 ± 0.04	− 0.08 ± 0.03	0.00 ± 0.02	0.07 ± 0.03	0.75	0.83	− 0.31	− 0.15	0.05	0.14	0.15	0.15	− 0.09
CT mineral %	0.14 ± 0.05	0.14 ± 0.04	− 0.09 ± 0.02	0.00 ± 0.03	0.07 ± 0.03	0.08 ± 0.01	0.8	0.03	− 0.15	0.1	0.15	0.15	0.17	− 0.05
CT phosphates/OM	0.15 ± 0.04	0.15 ± 0.04	− 0.10 ± 0.02	0.00 ± 0.02	0.08 ± 0.03	0.09 ± 0.02	0.11 ± 0.02	− 0.16	− 0.21	0.12	0.2	0.2	0.2	− 0.06
CT CO3/phosphates	0.00 ± 0.03	0.00 ± 0.03	0.00 ± 0.01	0.00 ± 0.02	0.00 ± 0.02	0.00 ± 0.02	0.00 ± 0.02	0.00 ± 0.03	0	0.11	0	0	0.01	0.11
MD OM %	− 0.25 ± 0.04	− 0.19 ± 0.04	0.12 ± 0.04	− 0.01 ± 0.03	− 0.08 ± 0.03	− 0.10 ± 0.03	− 0.12 ± 0.03	0.01 ± 0.03	0.21 ± 0.05	− 0.55	− 0.99	− 0.98	− 0.93	0.33
MD CO3%	0.13 ± 0.03	0.14 ± 0.04	− 0.06 ± 0.03	0.00 ± 0.01	0.04 ± 0.02	0.05 ± 0.01	0.06 ± 0.02	0.00 ± 0.02	− 0.11 ± 0.04	0.07 ± 0.02	0.56	0.5	0.52	0.42
MD phosphates %	0.24 ± 0.04	0.19 ± 0.04	− 0.12 ± 0.03	0.01 ± 0.03	0.08 ± 0.03	0.10 ± 0.03	0.12 ± 0.03	− 0.01 ± 0.03	− 0.21 ± 0.05	0.11 ± 0.04	0.20 ± 0.05	0.99	0.94	− 0.32
MD mineral %	0.25 ± 0.04	0.19 ± 0.04	− 0.12 ± 0.03	0.01 ± 0.02	0.08 ± 0.03	0.10 ± 0.03	0.12 ± 0.03	− 0.01 ± 0.02	− 0.20 ± 0.05	0.11 ± 0.03	0.20 ± 0.05	0.20 ± 0.05	0.94	− 0.38
MD phosphates/OM	0.26 ± 0.04	0.21 ± 0.04	− 0.13 ± 0.04	0.01 ± 0.03	0.08 ± 0.03	0.10 ± 0.04	0.13 ± 0.04	− 0.01 ± 0.03	− 0.22 ± 0.05	0.12 ± 0.04	0.21 ± 0.05	0.21 ± 0.05	0.22 ± 0.05	− 0.31
MD CO3/phosphates	− 0.11 ± 0.03	− 0.05 ± 0.03	0.06 ± 0.02	0.00 ± 0.00	− 0.05 ± 0.02	− 0.05 ± 0.01	− 0.06 ± 0.02	0.00 ± 0.01	0.08 ± 0.03	− 0.04 ± 0.01	− 0.08 ± 0.03	− 0.08 ± 0.03	− 0.09 ± 0.04	0.05 ± 0.03

TGA: thermogravimetry; OM: organic matter; CT: cortical bone; MD: medullary bone

Genetic correlations are estimated by multi-trait genomic restricted maximum likelihood, partial phenotypic correlations: phenotypes (adjusted for body weight) correlations

TGA measurements related to bone mineralization (Phosphates%, Mineral%, Phosphates/OM), either in the medullary or cortical bone showed positive genetic correlation estimates with tibia density and strength (Table 5). Conversely, TGA organic matter traits (Cortical and Medullary OM%) showed negative genetic correlation estimates with tibia density and strength. Such converse correlations are expected, because the mineral and organic component are the two main constituents of bone. Medullary CO3/Phosphates also showed a negative genetic correlation estimate with tibia density and strength traits.

For XRD mineral measurements (crystal scattering and orientations) in cortical bone, the estimated heritability and genetic correlations with tibia density and strength were very low (Table 6).

**Table 6:** Estimate of additive genetic variance (diagonal), genetic correlation (below diagonal) with standard errors, in addition to the partial phenotypic correlations (above diagonal), for XRD traits

	Tibia density	Tibia breaking strain	Crystal scattering	Crystal orientations	Crystal oriented fraction
Tibia density	0.44 ± 0.06	0.67	0.05	− 0.11	0.05
Tibia breaking strain	0.33 ± 0.05	0.42 ± 0.06	0.02	− 0.05	0.03
Crystal scattering	0.03 ± 0.03	− 0.02 ± 0.04	0.04 ± 0.05	− 0.03	− 0.04
Crystal orientations	− 0.06 ± 0.04	− 0.04 ± 0.04	0.00 ± 0.04	0.05 ± 0.04	− 0.21
Crystal oriented fraction	0.02 ± 0.03	0.03 ± 0.03	− 0.01 ± 0.03	0.00 ± 0.02	0.01 ± 0.03

XRD: X-ray diffraction; genetic correlations are estimated by multi-trait genomic restricted maximum likelihood; partial phenotypic correlations: phenotypes (adjusted for body weight) correlations

### Phenotypic correlations

The patterns for estimates of partial phenotypic correlations among traits (see Tables 4 and 5) were similar to those for estimates of genetic correlations, but they were higher in magnitude. For example, the estimated partial phenotypic correlation of cortical lipid with breaking strength was − 0.32, while the estimated genetic correlation was − 0.19.

## Discussion

In the present work, we combined different bone compositional measurements (FTIR and TGA) on tibia bone cortex and medulla with genotyping data to investigate the genetics of tibia bone characteristics in Rhode Island Red laying hens. Novel genetic markers associated with tibia composition (organic matter and mineral content) were detected. Among all the traits evaluated, the FTIR measurement of cortical lipid seems to be a key measurement since it had stronger significant genetic associations than the other traits, had quite a high estimate of heritability and was estimated to be genetically correlated with tibia density and strength. In this context, we will discuss the significant and suggestive genetic associations detected for the tibia composition traits, starting with the organic matter traits and then the mineral traits. Next, we will discuss the heritability estimates of tibia (cortical and medullary) composition traits and their genetic correlations with tibia density and strength.

### Genetic associations with organic matter traits

The results from the current study highlight the importance of FTIR cortical lipid measurement since, compared to all other FTIR traits, the cortical lipids showed the strongest associations and the highest genetic correlation estimates with tibia density and strength. This genetic correlation estimate was negative (− 0.20 ± 0.04), which suggests that lipid accumulation is related to a detrimental outcome for bone mechanical properties. Such negative genetic correlations are also reflected in the genome-wide association results, which detected six SNPs that had associations in opposite directions with tibia density and strength versus cortical lipids (Table 3). A decline in bone mass and an accumulation of adipocytes have been observed in mice with glucocorticoid-induced osteoporosis [30]. An inverse relationship between bone density and amount of adipose tissue was recently observed in both the femur and humerus bones of White Leghorn laying hens that suffer from bending/deviated keel bone [32]. The cells that underlie osteogenesis and adipogenesis share common bone marrow mesenchymal stromal progenitors [32, 33]. It is possible that certain hens have a genetic propensity that enhances stromal cell differentiation towards adipocytes, thereby reducing the number of mesenchymal progenitor cells that differentiate into osteoblasts. This potential mechanism is worthy of further investigation in laying hens, as it may underlie differences in bone strength. Low medullary mineralization in addition to the possible relationship between adipogenesis and osteogenesis could also be caused by depletion of medullary minerals towards eggshell formation [12, 13]. Previous results suggested that hens with stronger tibia bones have a greater medullary mineral content [13, 14], which is consistent with our findings. What is new in the current study is that a high level of mineralization in the tibial medullary bone was associated with lower lipid content in the tibial cortex bone.

Markers for cortical lipid associations overlap several compelling candidate genes for bone traits. The cortical lipid association on chromosome 2 (bp: 2,585,350) is located within the *WNT3A* gene (*Wnt family member 3A*), which encodes a cysteine-rich glycosylated protein that induces the expression of alkaline phosphate in bone mesenchymal cells [32, 33]. Alkaline phosphate is known as an osteoblastic mineralization marker, e.g. [34–36]. The WNT gene family is pivotal in regulating osteoblast differentiation and bone formation [37]. Loss of function of the WNT co-receptor LRP5 leads to decreased postnatal bone formation in both humans and mice [33], and a point mutation in this gene results in a high bone mass [38]. Due to linkage disequilibrium, these associations correspond to large regions of correlated markers that may overlap many genes. For example, nine markers in high linkage disequilibrium (${\mathrm{r}}^{2}$ > 0.8) with the lead SNP for cortical lipids on chromosome 2 (bp: 2,585,350), all together cover ~ 737 kb, and overlap the *WNT3A* and *WNT9A* genes in addition to other coding and non-coding sequences. Currently, we lack the genomic resolution to identify individual causative genes. Fine-mapping with sequence data might in the future provide better resolution for identifying the causative gene(s).

Two cortical lipid associations on chromosome 3 (bp: 27,548,492 and 27,648,733) are located within the *BRE* gene (*brain and reproductive organ-expressed*). Compared with normal bone, a seven-fold down regulation of *BRE* expression has been reported in osteoporotic human bone [39]. Knockdown of *BRE* in mouse bone marrow mesenchymal cells blocks the osteoblastic differentiation and enhances the expression of adipogenic marker genes, while its overexpression accelerates osteogenesis [40]. The cortical lipid association on chromosome 3 (bp: 27,351,346) is located within the *PPP1CB* gene (*protein phosphatase 1 catalytic subunit beta*), which encodes a protein involved in the molecular pathway for osteoclast proliferation and survival in humans [41]. All these previous findings suggest that lipid accumulation may promote osteoclast and suppress osteoblast proliferation. The SNPs that are in high linkage disequilibrium around the cortical lipid association on chromosome 3 cover ~ 444 kb and overlap with other genes in addition to *PPP1CB* and *BRE*, i.e. *TTC7A*, *CALM2*, *BRE,* and a gene of unknown function. The *CALM2* (*calmodulin 2*) gene encodes a protein that binds calcium and has been tied to bone function [42], so it also might be a candidate gene in that region.

The cortical lipid association on chromosome 2 overlaps with a suggestive locus for tibial cortical carbonate content in commercial laying hens [43]. The association with lipid content on chromosome 3 overlaps with a suggestive locus (p-value < 10^–4^) for cortical OM% in the current study (Table 3) and with comb weight in a study on crossed Beijing-You chicken [44].

In addition to the lipid associations, we detected associations with medullary CO3 1450/1415, which we hypothesize may be driven by differences in organic matter. This measurement represents the ratio between the peaks for carbonate (absorption peak: 1450 cm^−1^) and a secondary peak (1415 cm^−1^). In bone, the domain of the carbonate peaks (1400–1500 cm^−1^) overlaps with several absorption bands of proteins (CH, Amide II, COO−) or glycosaminoglycans (NH), as explained by Rey et al. [45]. We hypothesize that medullary CO3 1450/1415 is related to the medullary bone organic matter, given the strong positive phenotypic correlation (0.75) of medullary CO3 1450/1415 with medullary lipid. Still, the low genetic correlation estimate between that medullary organic matter and medullary CO3 1450/1415, and the lack of overlap of associated regions for these two traits suggest that their genetic basis may differ. The biological significance of these associations is an open question. Some markers that were found to be associated with medullary CO3 1450/1415 on chromosome 1 (bp: 175,454,102–176,773,112) overlap with a QTL for proventriculus weight that was detected in White Leghorn crossed with a Chinese indigenous line called Dongxiang Blue-Shelled [46]. The significant marker on chromosome 2 for medullary CO3 1450/1415 (bp: 176,597,999) overlaps with a QTL for blood total protein that was identified in Iranian broiler chickens [47]. Two significant markers associated with medullary CO3 1450/1415 (chromosome 1, bp: 176,670,270 and 176,699,327) are located within the *UBL3* gene (*ubiquitin-like 3 gene*), which encodes ubiquitin, a cell-level multifunctional signal [48]. Paget's disorder in humans, which causes bone tissue to be generated faster than normal, is caused by a mutation that impairs the binding of ubiquitin to a mediator of osteoclastogenesis [49, 50].

### Genetic associations with mineral traits

In the current study, we analysed bone mineral traits measured with the TGA and FTIR methods. In spite of quite high heritability estimates, the TGA measurements for bone mineral (and organic matter content) traits did not result in significant genome-wide associations. In contrast, FTIR measurements for bone mineral traits showed low genetic variation (average h^2^ ~ 0.07). When traits have a low heritability, more data are required to detect significant associations via GWAS, especially for highly polygenic traits. This could explain why the FTIR mineral traits showed fewer significant genetic associations than the FTIR organic matter traits, e.g. cortical lipid had a heritability estimate of 0.19.

Cortical crystal orientations displayed suggestive associations on chromosome 14. This component could be related to bone metabolism and/or turn-over rate since more mature bone shows greater crystal orientation in the mineral component [21]. This association overlaps with a QTL for wattle length in Beijing-You chicken [45], a QTL for 36-day body weight in Cobb-Vantress broiler [52], and a QTL for 21-day body weight in the slow-growing line selected by the SASSO breeding company [53].

### Bone composition heritabilities and genetic correlations

In the current study, estimates of heritability were based on the genomic relationship matrix, which is constructed using SNP genotypes and allele frequencies in the genotyped population. This approach reflects the genetic variance (and consequently the heritability) in the genotyped population rather than in the founder population, which is what is estimated using pedigree-based relationships [13]. For traits under selection, genetic variances decrease through generations, which is one reason why genomic-based heritability estimates may not be identical to the pedigree-based heritability estimates, such as those published by Dunn et al. [13] on the same Rhode Island Red population. For example, tibia density and strength had pedigree-based heritability estimates of 0.59 ± 0.09 and 0.51 ± 0.08, respectively, in Dunn et al. [13], but a genomic-based heritability estimates of 0.50 ± 0.05 and 0.46 ± 0.05, respectively, in the current study.

Genomic heritability estimates for FTIR measurements (Table 1) suggest that the traits with the highest genetic variability in tibia composition are related to organic matter, in particular cortical lipid (the highest FTIR h^2^: 0.20 ± 0.05). However, the heritability estimates for TGA measurements suggest that the traits with the highest genetic variability in tibia composition are medullary OM%, followed by medullary phosphate%, cortical OM%, and cortical phosphate%. The discrepancy between FTIR and TGA heritability estimates may be due to different principles underlying these two methods, which probably reflect similar but not identical components. For example, FTIR measures lipids alone, while TGA measures all the organic matter without discriminating lipids.

In general, estimates of genetic correlations between bone composition and mechanical (density and strength) traits were not strong, less than 0.25 (see Tables 4 and 5). This aligns with an earlier study that reported low phenotypic correlations of tibia FTIR mineralization traits with tibia density and breaking strength in caged White Leghorn birds [22]. One methodological difference that may contribute to a low correlation is that bone composition traits are measured locally at the tibia mid-shaft, while density and strength traits are measured on the whole tibia. In line with previous papers [22, 27, 51, 52], the estimated genetic correlations suggest that both the cortical and medullary bones contribute to bone density and strength traits, and contributions from the cortex are greater than those form the medullary bone, because the genetic correlations are higher for the former.

An earlier study reported a very low positive phenotypic correlation of cortical mineralization traits (PO4/Amide I and CO3/Amide I) with tibia density and strength [22], while in our study the estimate of the genetic correlation between these two traits was negative. A negative relationship between cortical mineralization and bone strength appears paradoxical but could be explained by indirect relationships. If there is low genetic variation in the numerators (representing phosphate and carbonate) then the variability of the ratios PO4/Amide I and CO3/Amide I, could be driven by the variability of the denominator representing organic matter. The organic matter, in both cortical (Table 5) and medullary [13] bones, tends to correlate negatively with bone density and strength.

On the other hand, medullary mineralization (PO4/Amide I and CO3/Amide I) had positive genetic correlation estimates with tibia density and strength and the heritability estimates were higher for the medullary than for the cortical mineralisation traits (as measured by TGA), which suggests the importance of the medullary mineral phase for bone strength. These same genetic relationships were also observed by Dunn et al. [13] with pedigree-based estimates. However, these medullary mineralization traits are genetically negatively correlated with average egg mass in the same Rhode Island Red population, as shown previously by Dunn et al. [13]. Laying larger eggs may be the mediating factor between bone damage issues and the egg laying process in Rhode Island Red laying hens and, thus, genetic selection for slightly smaller eggs may improve bone strength.

Cortical carbonate substitution (Cortical CO3/PO4) has been related to cortical bone mineral turnover [22]. Bone turnover has two dimensions: resorption and deposition. Because cortical CO3/PO4 correlates negatively with cortical mineralization but positively with medullary mineralization, we hypothesize that mineral resorption from the tibial cortex is associated with deposition (or mineralization) in the tibial medulla. This may explain the positive genetic correlation estimate of cortical carbonate substitution with tibia strength as does the resorption and deposition on the same tibia bone i.e. the mineral that has been resorbed from the tibial cortex is perhaps deposited on tibial medulla. However, medullary carbonate substitution had negative or zero genetic correlation estimates with tibia strength, probably because the mineral that was resorbed from the tibial medullary bone is deposited somewhere else rather than the tibia bone e.g. eggshell [12, 13].

Our genetic correlation estimates between cortical and medullary FTIR measurements were similar to the respective phenotypic correlations reported in caged White Leghorn [22], where the positive correlation between mineralization traits (PO4/Amide I and CO3/Amide I) indicated that CO3 and PO4 levels share a genetic basis. In addition, these mineralization traits correlate negatively with carbonate substitution (CO3/PO4). The lower carbonate substitution in bone minerals is, the more the minerals mineralize the organic matter, which indicates more matured bones.

## Conclusions

The present study detected novel genetic associations for bone composition traits, in particular for organic matter, which could be used as a basis for further molecular genetics and functional investigations. Among all FTIR traits, cortical lipids displayed the strongest genetic associations among all FTIR and TGA traits and the strongest genetic correlations with tibia density and strength. Our results also highlight cortical bone lipid content as a key measurement for further genetic or non-genetic avian bone studies.

## Supplementary Information

**Additional file 1: Table S1.** Significant SNPsper trait with their positions, estimated marker effect and p-value.

**Additional file 2: Table S2.** Overlap of associationsdetected in the current study and associations or QTL in Chicken QTLdb.

**Additional file 3: Figure S1.** Heat map showing the linkage disequilibriumbetween all SNPs detected with a significant level lower than 10^–4^. Linkage disequilibrium statistics ${\mathrm{r}}^{2}=({{\mathrm{P}}_{AB}-{\mathrm{P}}_{A}{\mathrm{P}}_{B})}^{2}/({\mathrm{P}}_{A}{\mathrm{P}}_{B}{\mathrm{P}}_{a}{\mathrm{P}}_{b})$ where $\mathrm{P}$ is the frequency, $A/a$ is the first/second allele at a given locus and $B/b$ is the first/second allele at another locus.

## Data Availability

The datasets analysed during the current study containing genotypes information are not publicly available due to commercial sensitivity. The phenotypes data has been archived as mentioned in [13].
