# Supplementary figures and images for: Genetic markers associated with bone composition in Rhode Island Red laying hens
Source: Genet Sel Evol. 2023 Jun 29;55:44. doi: 10.1186/s12711-023-00818-x (PMC10311847; doi:10.1186/s12711-023-00818-x)

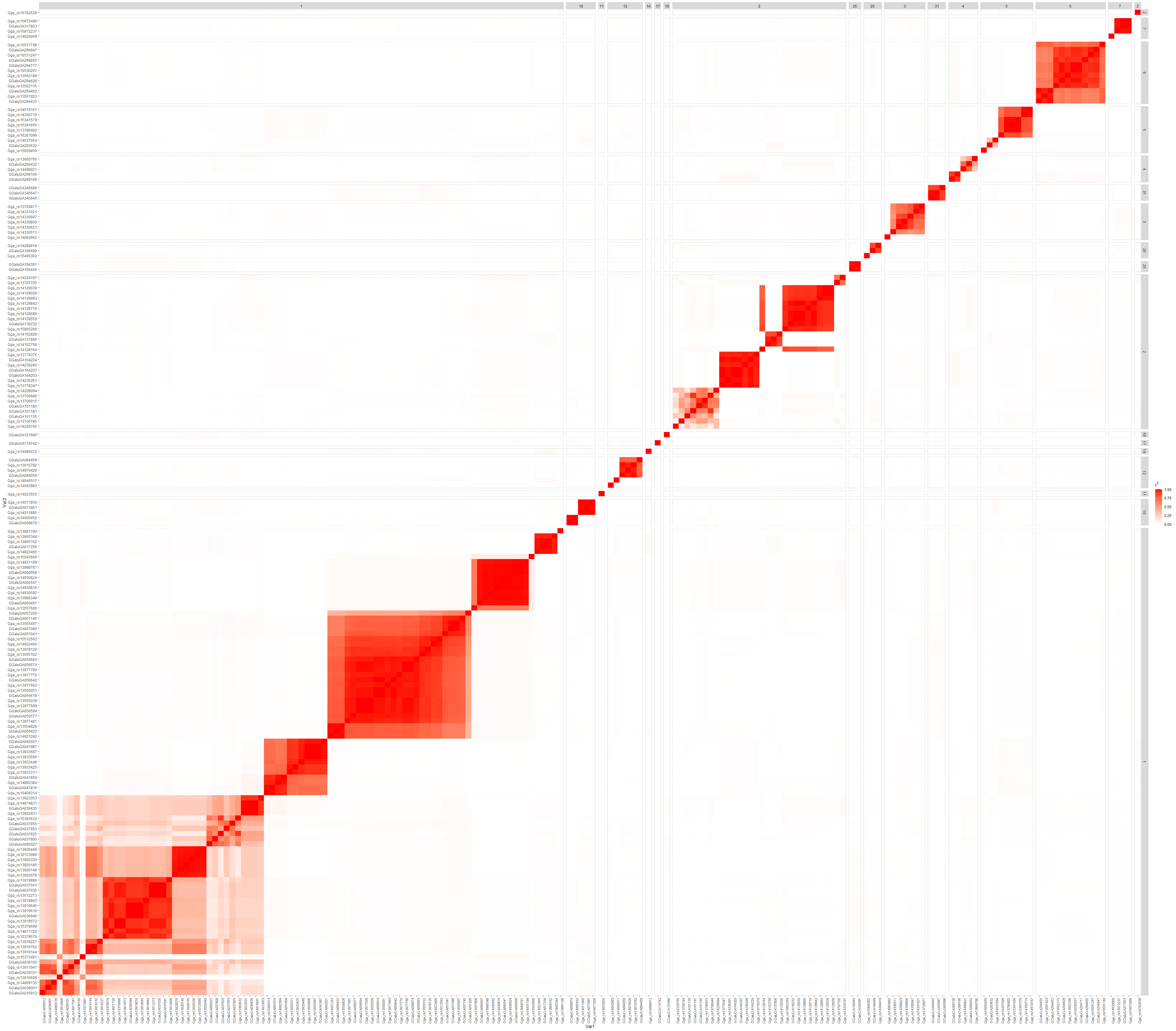

Supplement: Supplementary file 3 — Additional file 3: Figure S1. Heat map showing the linkage disequilibriumbetween all SNPs detected with a significant level lower than 10–4. Linkage disequilibrium statistics ${\mathrm{r}}^{2}=({{\mathrm{P}}_{AB}-{\mathrm{P}}_{A}{\mathrm{P}}_{B})}^{2}/({\mathrm{P}}_{A}{\mathrm{P}}_{B}{\mathrm{P}}_{a}{\mathrm{P}}_{b})$r2=(PAB-PAPB)2/(PAPBPaPb) where $\mathrm{P}$P is the frequency, $A/a$A/a is the first/second allele at a given locus and $B/b$B/b is the first/second allele at another locus. [file 12711_2023_818_MOESM3_ESM.png]
